# High Prevalence of Antibiotic-Resistant Gram-Negative Bacteria Causing Surgical Site Infection in a Tertiary Care Hospital of Northeast India

**DOI:** 10.7759/cureus.12208

**Published:** 2020-12-21

**Authors:** Sangeeta Deka, Deepjyoti Kalita, Putul Mahanta, Dipankar Baruah

**Affiliations:** 1 Medical Microbiology, Fakhruddin Ali Ahmed Medical College and Hospital, Barpeta, IND; 2 Medical Microbiology, All India Institute of Medical Sciences, Rishikesh, IND; 3 Microbiology, All India Institute of Medical Sciences, Rishikesh, IND; 4 Forensic Medicine, Assam Medical College and Hospital, Dibrugarh, IND; 5 Pathology, Fakhruddin Ali Ahmed Medical College, Barpeta, IND

**Keywords:** ssi, antibiotic resistance, microbiology, healthcare-associated infections, aerobic bacteria

## Abstract

Background and objective

Surgical site infections (SSI) are the most common healthcare-associated infections in low- and middle-income countries associated with substantial morbidity and mortality and impose heavy demands on healthcare resources. We aimed to study the microbiological profile of SSI pathogens and their antibiotic-resistant patterns in a tertiary care teaching hospital serving mostly rural population

Methods

A prospective, hospital-based cross-sectional study on pathogen profile and drug resistance was conducted from January 2015 to December 2016. Study subjects were the patients who developed signs of SSI after undergoing surgical procedures at three surgical wards (General Surgery, Orthopedics, and Obstetrics & Gynecology). The selection of the patients was based on CDC Module. Standard bacteriological methods were applied for isolation of pathogens and antibiotic-susceptibility testing based on CLSI (Clinical Laboratory Standard Institute) guidelines.

Results

Out of 518 enrolled subjects, 197 showed growth after aerobic culture yielding 228 pathogen isolates; 12.2% of samples showed polymicrobial growth. *Escherichia coli* (22.4%) and Klebsiella species (20.6%) were the predominant isolated bacteria followed by Staphylococcus species (18.4%), Pseudomonas species (12.3%), and Enterococcus species (6.6%). Gram-negative bacteria (GNB) were highly resistant to ampicillin (90.1%) and cefazolin (85.9%). High resistance was also observed to mainstay drugs like ceftriaxone (48.4%), cefepime (61%), amoxycillin-clavulanic acid (43.4%), and ciprofloxacin/levofloxacin (37.7%). Among the Gram-positive cocci, *Staphylococcus aureus* showed 85-96% resistance to penicillin and 65-74% to ampicillin. But GPCs were relatively less resistant to quinolones (16-18%) and macrolides (21.5%). *S. aureus* was 100% sensitive to vancomycin and clindamycin but vancomycin-resistant Enterococci was encountered in 3/15 (20%) isolates.

Conclusion

GNBs were responsible for more than two-thirds of aerobic-culture positive SSI and showed high resistance to the commonly used antibiotics thus leaving clinicians with few choices. This necessitates periodic surveillance of causative organisms and their antibiotic-susceptibility pattern to help in formulating hospital antibiotic policy. The antibiotic stewardship program is yet to be adopted in our hospital.

## Introduction

Modern surgery has proved to be a great boon to mankind which took its roots in the nineteenth century after Lister introduced the aseptic treatment of wounds. Following the discovery of antibiotics in the twentieth century, it was further revolutionized, when complicated, life-saving, and reconstructive surgeries became possible. But following the emergence of drug-resistant organisms, surgical site infections (SSI) posed a major challenge to the advancement in the surgical field.

An SSI is an infection that occurs after surgery, in the part of the body where the surgery took place, within 30 days of operation or after one year if an implant is placed [1,2]. It can range from superficial infections involving the skin only to severe forms involving tissues under the skin, organs, or implanted material [1,2]. SSIs are classified into incisional SSIs, which can be superficial or deep, and organ/space SSIs, which affect the rest of the body other than the body wall layers [2].

SSIs are the most frequent of all healthcare-associated infections (HCAI) in low- and middle-income countries (LMICs) and second most frequent in Europe and the USA and are a major contributor to morbidity and mortality due to HCAI [3,4]. The proportion of SSI was reported to be as high as 29% of all health-care-associated infections [4]. Incidence rates of SSI in LMICs vary widely ranging from 1.2 to 23.6 per 100 surgical procedures and despite the high level of heterogenicity in studies from LMICs, the pooled incidence was estimated at 11.8% [3-5]. About 39-51% of pathogens causing SSI in the USA were documented to be resistant to standard prophylactic antibiotics [5]. But robust data defining the burden of SSI and the pattern of micro-organisms responsible for causing SSI in LMICs remain scarce compared to high-income countries [3,4,6]. Moreover, the suspected estimate of cases can be higher as many minor cases are unreported and undertreated. SSI surveillance and timely feedback of results are strongly recommended by the World Health Organisation as part of the core components of effective infection prevention and control programs. Conducting regular and high-quality SSI surveillance is crucial in the preparation of hospital antibiotic policy and monitoring strict adherence to it [5]. Cases getting inadequate antimicrobial treatment (mostly with a combination of wide-spectrum antibiotics with inadequate dosing) pose a threat to the community as it may result in the emergence of multi-drug resistant strains of bacteria [7].

The objectives of the study were to show the distribution of SSI in a tertiary care center of northeast India serving mostly rural population of lower Assam, over a two years period; and to study the microbiological profile of SSI pathogens and their antibiotic-resistant patterns, isolated from three surgical wards.

## Materials and methods

This is a hospital-based cross-sectional study, conducted over a period of two years from January 2015 to December 2016 at a tertiary care teaching hospital. The study was approved after it was ethically reviewed by the Institutional Ethics committee of our institute (FAAMCH/Ethical Committee/128/2012/5704; dated 4.12.2015). All surgical patients admitted in the Department of General Surgery, Obstetrics & Gynecology, and Orthopedics department, who had been operated on during the study period and had developed any signs of SSI during the period of hospitalization was considered to be eligible for the study. Any patients who had presented the clinical signs of SSI like purulent drainage from the superficial incision, developed localized pain or tenderness, localized swelling, erythema, malodor or heat, or had developed fever within 30 days of surgery were included in the study [8]. Surgical wounds were divided into clean, clean-contaminated, contaminated, and dirty (CDC classification), based on the type of surgery and intraoperative events mentioned in the surgeon’s operation and anesthesia notes in the patient’s medical chart. Patients with incomplete data were excluded from the study [1]. Patients with minor day-care surgeries were also excluded from the study.

Two swabs were collected from the visibly infected area of the subjects by gently introducing them into the wound sites and rotating the swab tips in the wound, taking care to avoid contamination of specimens with commensals from the skin. These swabs were sent immediately to the Microbiology Laboratory of our institute in sterile containers. The swabs were inoculated in blood agar, MacConkey agar, and Sabouraud dextrose agar media and incubated aerobically at 37°C. Plates were checked for growth of organisms after 24 hours (overnight) and 48 hours of incubation. If there was any visible growth, it was identified by standard phenotypic methods and was subjected to antibiotic-susceptibility testing (Kirby-Bauer disc diffusion method) as described in the CLSI (Clinical Laboratory Standard Institute) guidelines [9]. Socio-demographic data and clinical information concerning all patients with wound infections after surgery were retrieved from the medical records maintained in the department.

The collected data were entered into a Microsoft Excel spreadsheet by two researchers independently. Each chart was manually reviewed and checked for accuracy by the third researcher. Tables and graphs were prepared using MS office and Excel table functions. Descriptive statistics like arithmetic mean were calculated using the Statistical Package for Social Sciences (SPSS) version 23 (IBM Corp., Armonk, NY).

## Results

Sociodemographic characteristics

Samples of 518 included subjects, who underwent surgery and had evidence of SSI, were tested for the presence of micro-organisms. The age of the included cases ranged from 15 years to 71 years with a mean age of 31.57 years; 56.56% (n=293) cases were females while the rest 43.44% (n=225) were males. The mean age in the case of females was 29.4 years but the mean age for males was higher at 37.6 years. The majority of the cases (86.1%) came from rural areas while 13.9% dwelled in towns. Co-morbid health conditions like diabetes mellitus, hypertension, bronchial asthma, cardiovascular disease, HIV/AIDS, other infections, multiple co-morbidities, etc. were found to be associated with 28.38% of cases mostly in the age group of >50 years. Among the co-morbid conditions, hypertension (28.6%) and diabetes mellitus (26.5%) were the most common. The majority of the procedures had non-clean wounds, mainly contaminated (34%) and clean-contaminated (30%). Only 9% of the clean wounds developed symptoms of SSI (Table 1).

**Table 1:** Sociodemographic and clinical characteristics of patients at surgical wards (N=518) *Cardiovascular disease 16 (10.9%); hypertension 42 (28.6%); diabetes mellitus 39 (26.5%); HIV/AIDS 4(2.7%); other infections 14 (9.5%); bronchial asthma 16 (10.9%); multiple co-morbidities 7 (4.8%); others 9 (6.1%). The majority of the procedures had non-clean wounds, mainly contaminated (34%) and clean-contaminated (30%).

Characteristics		N	%
Age	<30 years	167	32.24
	30-50 years	202	39.00
	>50 years	149	28.76
Gender	Male	225	43.44
	Female	293	56.56
Place of Residence	Rural	446	86.10
	Urban	72	13.90
Religion	Hindu	181	34.94
	Muslim	334	64.48
	Others	3	0.58
Co-morbidity*	Present	147	28.38
	Absent	371	71.62
Admission	ICU/semi ICU	77	14.86
	Wards	441	85.14
Wound class	Clean	47	9.07
	Clean contaminated	154	29.73
	Contaminated	176	33.98
	Dirty	141	27.22

Types of surgeries conducted in patients who had developed signs of SSI are tabulated in Table 2. In males, the majority of the cases followed orthopedic surgeries (33.8%) like an open reduction of fractures and multiple fractures in road traffic accidents. While in females almost one-third (33.1%) of the cases occurred after cesarean section. Both upper and lower gastrointestinal surgeries followed next in both the gender; however, a number of cases were more in males than in females (26.2% versus 17.7% in hepatobiliary surgeries and 12% versus 10.6% in appendicectomy).

**Table 2:** Frequency distribution of different types of surgery done to female and male patients under the study GI: gastrointestinal; GB: gall-bladder; RTA: road-traffic accident; ORIF: open reduction and internal fixation

Department	Type of surgery	Female n,%	Male n,%	Total n,%
General Surgery	Cholecystectomy, choledocholithotomy, choledochal cyst excision Liver resection, or other bile ducts/GB related operations	52 (17.7)	59 (26.2)	111 (21.4%)
	Appendicectomy	31 (10.6)	27 (12)	58 (11.2)
	Hepatico-jejunostomy, gastrectomy, gastrojejunostomy, truncal vagotomy, hernia	12 (4.1)	20 (8.9)	32 (6.2)
	Other surgeries (lipoma, cyst excision, breast surgeries, etc.)	11 (3.8)	9 (4)	20 (3.9)
Obstetrics & Gynecology	Caesarean section	97 (33.1)	0	97 (18.7)
	Other gynecological surgeries (hysterectomy, myotomy, colporrhaphy, etc.)	39 (13.3)	0	39 (7.5)
Orthopedics	Fractures (ORIF)/RTA	25 (8.5)	76 (33.8)	101 (19.5)
	Other orthopedic surgery (arthroplasty)	28 (9.6)	32 (14.2)	60 (11.6)
Total		293	225	518

Microbiological findings

Out of the 518 samples, 197 samples showed growth of microorganisms (bacteria, n=189; fungi, n=12; 4 samples showed growth of both bacteria and fungus) after aerobic culture at 37°C (38% aerobic culture positivity). The frequency of occurrence of different pathogens is shown in Table 3. Single bacterial isolates were recovered from 173 samples (87.8%) whereas 24 cases (12.2%) had polymicrobial infections. Thus, 197 culture-positive samples yielded a total of 216 bacterial isolates and 12 fungal isolates (total 228 isolates). More than two-thirds of the isolates were Gram-negative bacilli (GNB) (n=151, 69.74%) (Table 3). Escherichia coli was the predominant isolates (22.37%) followed by Klebsiella spp. (20.61%). Among the Gram-positive cocci (GPC) Staphylococcus spp. constituted 18.42% (n: 23+19=42).

**Table 3:** Frequency of the microorganisms isolated from wound cultures GNB: Gram-negative bacilli; GPC: Gram-positive cocci; CONS: coagulase-negative Staphylococci; spp.: species *Calculated based on a total of 228 identified pathogens; (in 24 wounds more than one pathogen was isolated).

Type of microorganism	Frequency (N)	(%)*
Total Gram-negative bacilli	159	69.74
Escherichia coli	51	22.37
Klebsiella spp.	47	20.61
Enterobacter spp.	9	3.95
Proteus species	7	3.07
Pseudomonas aeruginosa	28	12.28
Acinetobacter spp.	10	4.39
Other GNBs	7	3.07
Total Gram-positive cocci	65	28.51
Staphylococcus aureus	23	10.09
CoNS	19	8.33
Enterococcus spp.	15	6.58
Streptococcus spp.	8	3.51
Fungi	12	5.26
Candida spp.	11	4.82
Other fungus	1	0.44

Resistance pattern of the isolated organisms

**Figure 1:**
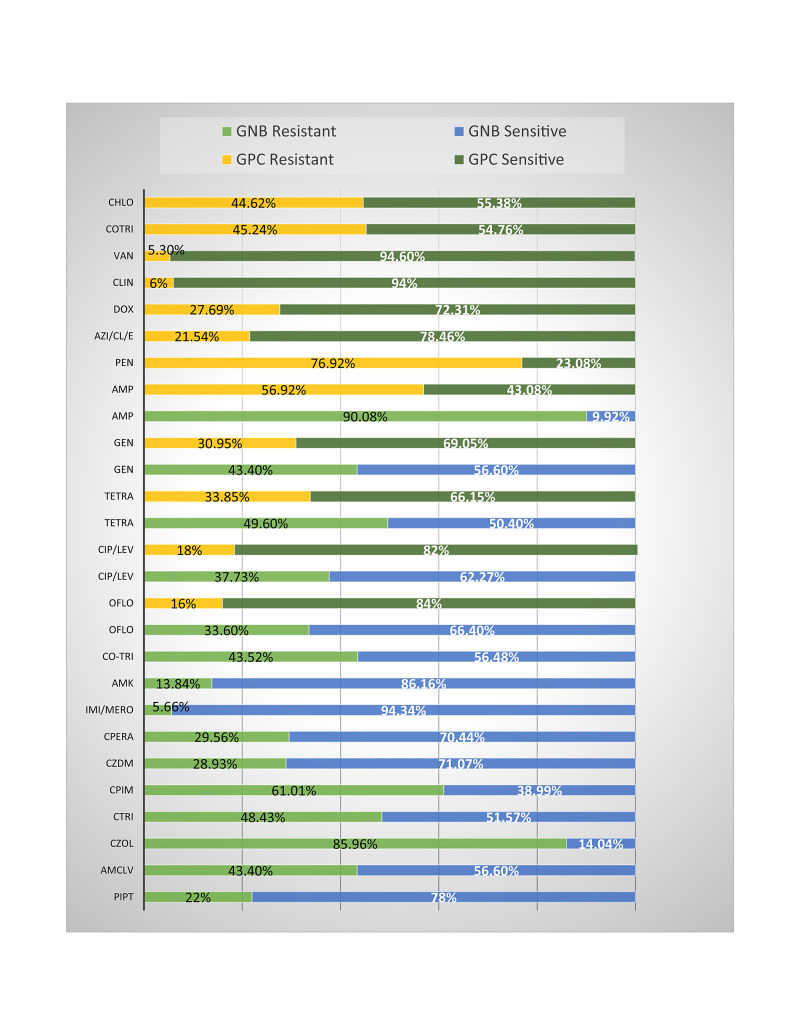
Overall antibiotic resistance pattern of Gram-negative and Gram-positive bacteria causing surgical site infections Note: Gram-negative bacteria include *E. coli*, Klebsiella spp, Pseudomonas spp., Acinetobacter spp., Enterobacter spp, Proteus spp., and some other Gram-negative bacilli; Gram-positive bacteria includes Staphylococcus spp., Enterococcus spp., and Streptococcus spp. Amp: ampicillin; PipT: piperacillin-tazobactum; AmClv: amoxicillin-clavulanic acid; Czol: cefazoline; Ctri: ceftriaxone; Cpim: cefepime; Czdm: ceftazidime; Cpera: cefoperazone; Imi: imipenem; Mero: meropenem; Gen: gentamicin; Amk: amikacin; Tetra: tetracycline; Cip: ciprofloxacin; Lev: levofloxacin; Mox: moxifloxacin; Oflo: ofloxacin; Cotri: trimethoprim-sulfamethoxazole; CoNS: coagulase negative Staphylococcus; Pen: penicillin; Azi: azithromycin; Cl: clarithromycin; E: erythromycin; Dox: doxycycline; Clin: clindamycin; Chlo: chloramphenicol; Van: vancomycin

Drug resistance of overall Gram-negative bacilli irrespective of species was 90.1% to ampicillin, 22% to piperacillin-tazobactam, 43.4% to amoxicillin-clavulanic acid, 85.9% to cefazoline, 48.4% to ceftriaxone, 61% to cefepime 28.9%, to ceftazidime, 29.6% to cefoperazone, 5.7% to imipenem/meropenem, 43.4% to gentamicin; 13.8% to amikacin, 49.6% to tetracycline, 37.7% to ciprofloxacin/levofloxacin and ofloxacin, 43.5% to trimethoprim-sulfamethoxazole (Figure 1). Resistance patterns exhibited by individual species are listed in Table 4.

**Table 4:** Antimicrobial resistance pattern in Gram-negative bacilli in 159 isolates causing surgical site infections Amp: ampicillin; PipT: piperacillin-tazobactum; AmClv: amoxicillin-clavulanic acid; Czol: cefazoline; Ctri: ceftriaxone; Cpim: cefepime; Czdm: ceftazidime; Cpera: cefoperazone; Imi: imipenem; Mero: meropenem; Gen: gentamicin; Amk: amikacin; Tetra: tetracycline; Cip: ciprofloxacin; Lev: levofloxacin; Mox: moxifloxacin; Oflo: ofloxacin; Cotri: trimethoprim-sulfamethoxazole *Other GNBs include Citrobacter spp., Serratia spp. (2 isolate each), *Morganella morganii*, Burkholderia spp, and *Alcaligenes faecalis* (1 isolate each, respectively). **Percentage calculated based on the number of isolates resistant to an antibiotic out of the total isolates subjected to susceptibility testing to that particular antibiotic.

	Escherichia coli n (%)	Klebsiella spp. n (%)	Pseudomonas spp. n (%)	Acinetobacter spp. n (%)	Enterobacter spp. n (%)	Proteus spp. n (%)	Other GNBs n (%)*	Overall GNB (%)**
Amp	47 (92.2)	45 (95.7)	-	-	8 (88.9)	5 (71.4)	4 (57.1)	90.1
PipT	11 (21.6)	9 (19.1)	9 (32.1)	3 (30)	1 (11.1)	0 (0)	2 (28.6)	22.0
AmClv	21 (41.2)	18 (38.3)	17 (60.7)	6 (60)	2 (22.2)	3 (42.8)	2 (28.6)	43.4
Czol	42 (82.4)	44 (93.6)	-	-	5 (55.6)	7 (100)	-	85.9
Ctri	24 (47.1)	20 (42.6)	17 (60.7)	7 (70)	0 (0)	4 (57.1)	5 (71.4)	48.4
Cpim	35 (68.6)	31 (65.9)	14 (50)	6 (60)	3 (33.3)	3 (42.8)	5 (71.4)	61.01
Czdm	15 (29.4)	10 (21.3)	12 (42.8)	5 (50)	0 (0)	1 (14.3)	3 (42.8)	28.9
Cpera	17 (33.3)	14 (29.8)	9 (32.1)	6 (60)	0 (0)	0 (0)	1 (14.3)	29.6
Imi/Mero	3 (5.9)	1 (2.1)	2 (7.1)	3 (30)	0 (0)	0 (0)	0 (0)	5.7
Gen	23 (45.1)	22 (46.8)	12 (42.8)	3 (30)	3 (3.33)	2 (28.6)	4 (57.1)	43.4
Amk	5 (9.8)	4 (8.5)	7 (28)	2 (20)	1 (11.1)	1 (14.3)	2 (28.6)	13.8
Tetra	26 (51)	22 (46.8)	-	4 (40)	4 (44.4)	4 (57.1)	5 (71.4)	49.6
Cip/Lev	20 (39.2)	19 (40.4)	11 (39.3)	4 (40)	2 (22.2)	2 (28.6)	2 (28.6)	37.7
Ofl	19 (37.3)	13 (27.7)	9 (32.1)	-	2 (22.2)	3 (42.8)	4 (57.1)	33.6
Co-tri	19 (37.3)	21 (44.7)	-	6 (60)	4 (44.4)	4 (57.1)	-	43.5

Among GPC isolates, maximum resistance was observed to penicillin (76.9) and ampicillin (56.9%) followed by trimethoprim-sulfamethoxazole (45.2%), chloramphenicol (44.6%), tetracycline (33.8%), gentamicin (30.9%), doxycycline (27.7%), the macrolides (21.5%), the quinolones (18.0%), vancomycin (5.3%), and clindamycin (6%). Individual species drug resistance patterns are given in Table 5. A comparison of the resistance pattern exhibited by Gram-positive bacteria and Gram-negative bacteria is depicted in Figure 1.

**Table 5:** Antimicrobial resistance pattern of important Gram-positive cocci in 65 isolates causing surgical site infections CoNS: coagulase-negative Staphylococcus; Pen: penicillin; Amp: ampicillin; Gen: gentamicin; Azi: azithromycin; Cl: clarithromycin; E: erythromycin; Tetra: tetracycline; Dox: doxycycline; Cip: ciprofloxacin; Lev: levofloxacin; Mox: moxifloxacin; Oflo: ofloxacin; Clin: clindamycin; Cotri: trimethoprim-sulfamethoxazole; Chlo: chloramphenicol; Van: vancomycin *Percentage calculated based on the number of isolates resistant to an antibiotic out of the total isolates subjected to susceptibility testing to that particular antibiotic.

	Staphylococcus aureus n (%)	CoNS- n (%)	Enterococcus spp. n (%)	Streptococcus spp. n (%)	Overall GPCs*
Pen	20 (86.96)	18 (94.74)	10 (66.67)	2 (25)	76.9
Amp	15 (65.23)	14 (73.68)	8 (53.33)	0	56.9
Gen	9 (39.13)	4 (21.05)	-	-	30.9
Azi/Cl/E	5 (17.85)	3 (15.79)	5 (33.33)	1 (12.5)	21.54
Tetra	6 (26.09)	7 (36.84)	7 (46.67)	2 (25)	33.8
Dox	5 (21.74)	7 (36.84)	6 (40)	0	27.7
Cip/Lev/Mox	5 (21.74)	4 (21.05)	2 (13.33)	1 (12.5)	18.46
Oflo	5 (21.74)	2 (10.53)	-	1 (12.5)	16
Clin	0 (0)	3 (15.79)	-	0 (0)	6
Cotri	10 (43.48)	9 (47.36)	-	-	45.2
Chlo	11 (47.83)	10 (52.63)	4 (26.67)	4 (50)	44.6
Van	0	0	3 (20)	-	5.3

## Discussion

Despite improvements in infection control measures, post-surgical infection of wounds remains a significant problem that could be associated with any surgical procedures but commoner in contaminated surgeries as was observed in our current work. Studies show that the incidence of SSI in hepato-biliary, colo-rectal surgeries, cesarean section, and orthopedic surgeries is higher [4-6,10]. Gender differences in SSI exist and are procedure-specific [11]. We found a lesser number of females to be affected in orthopedic, biliary, and gut surgeries compared to males.

The present study observed an aerobic culture positivity rate of 38% and multiple pathogens were found to be implicated in causing SSI. The most striking finding of the current study was that almost half of the isolates belonged to Enterobacteriaceae. *E. coli* and Klebsiella spp. were found to be the commonest organisms and together constituted 43% of the total isolates. *Staphylococcus aureus* has been documented as the commonest pathogen causing SSI with rates as high as 30.4% [3,12,13]. Few other studies from India also reported *S. aureus* as the commonest isolate [14,15]. But some studies corroborate our findings. Borse et al., Dessie et al., and Amare et al. found *E. coli* to be the commonest organism (32.58%, 23.1%, and 24.3%, respectively) causing SSI [16-18]. The predominance of GNBs in our study which covered almost two-thirds of the total isolates points towards a difference in organism profile in LMIC compared to high-income countries, especially in resource-scant healthcare facilities. Poor knowledge of personal hygiene of the patients, high environmental burden of GNBs, inadequate infection control practices like especially in the post-surgical wards might play an important role in the development of SSI. Moreover, increased rates of intrinsically resistant bacteria like Acinetobacter and Pseudomonas (together 16.6% of isolates) are a major concern. In many cases of SSI, the responsible pathogen arises from the patient’s own endogenous flora (both anaerobic and aerobic). But pathogens might also originate from exogenous sources like incompletely sterilized devices or instruments, the caregivers, etc. (mostly comprises aerobic GPC like* Staphylococcus* spp.) [12]. *E. coli* has been implicated as a major pathogen in abdominal surgeries [19,20], and the source can be both endogenous (patient’s gut flora) or exogenous (unhygienic surroundings, poor personal hygiene, or post-procedural contamination) [12,21]. Source of non-fermenters like Acinetobacter and Pseudomonas is usually exogenous like hospital surroundings or contaminated devices or dressings [12]. Overall Gram-negative bacilli are also more resistant to disinfectants compared to Gram positives [19,21]. The greater complexity of the structure of the GNB cell wall renders intrinsic resistance to most disinfectants and antibacterial agents used in the hospitals. The efficacy of the commonly used disinfectants and antiseptics towards resistant organisms is another area that needs to be explored.

In-vitro antibiotic susceptibility testing revealed that the isolated bacteria reacted differently to different antibiotics. Enterobacteriaceae like *E. coli* and Klebsiella showed very high resistance to ampicillin (92.2% and 95.7%) and first-generation cephalosporins like cefazolin (82.4% and 93.6%) similar to findings of other studies [13,19,20]. They also showed high resistance to amoxycillin-clavulanic acid (38-43%), gentamicin (45-48%), tetracycline (46-51%), and co-trimoxazole (37-45%). High resistance exhibited to third-generation cephalosporins like ceftriaxone (42-48%) and quinolones like ciprofloxacin and levofloxacin (39-41%) is a matter of grave concern as they are widely used as mainstay drugs and leave clinicians with few choices. Striking resistance of 65-69% to fourth-generation cephalosporins like cefepime is alarming. High resistance to many of these drugs was also reported by other studies conducted elsewhere [18,20,21,22].

Another matter of concern is the emergence of glucose non-fermenting bacteria like Pseudomonas and Acinetobacter as causative agents of SSI as they are intrinsically resistant to many antibiotics. In this study, they showed high resistance to amoxycillin-clavulanic acid (60%), ceftriaxone (60-70%), cefepime (50-60%), gentamicin (32-43%), and quinolones (40%). Even anti-Pseudomonal drugs like ceftazidime and cefoperazone showed high resistance of 42-50% and 32-60%, respectively. A similar concern was also expressed by other authors [23,24].

Among the GPCs, Staphylococcus spp. (*S. aureus* and CoNS) showed very high resistance to penicillin (87-95%) and ampicillin (65-74%). However, low resistance was reported against clindamycin (0-15%) and macrolides (15-17%). Isolated Staphylococcus spp. showed 100% susceptibility to vancomycin. Isolation of another GPC Enterococci spp. which is also a normal gut-flora, again points towards fecal contamination due to poor hygiene. Vancomycin-resistant Enterococci were detected in three out of 15 isolates (20%). However, compared to GNBs, the GPCs were relatively more susceptible to quinolones, gentamicin, and tetracycline (Figure 1). A similar study from Kolkata observed a decreasing trend of drug-resistant Staphylococcus and reported low resistance to quinolones and doxycycline and 100% sensitivity to vancomycin, linezolid, and tigecycline [23].

Given these results, an effective antibiotic stewardship program with an evidence-based antibiotic policy is very much essential in our hospital. Unfortunately, being a relatively new hospital, such a program is yet to be adopted. We expect that our current work will help in initiating this program in our hospital. 

As there is limited local and regional data on the resistance pattern of pathogens causing SSI, this report can serve as a unique benchmark for caregivers engaged in SSI prophylaxis and formulating antimicrobial stewardship programs. However, limitation of the study is that sensitivity patterns to important antibiotics like tigecycline and cefotaxime could not be ascertained due to irregular availability of data. Also, the molecular characterization of the multidrug-resistant organisms could not be done due to the unavailability of resources and required infrastructure. As a future scope, study including more identical subjects (e.g., GI surgery or Gynaecologic surgery or orthopedic post operatives separately) would make the analysis more homogenous, and to be taken up as follow-up work. Further[L1] study evaluating the determinants and predictors that leads to the development of antibiotic resistance and strategies to control them is necessary to determine their impact on patient outcomes.

## Conclusions

Despite the increased use of minimally invasive surgery and enhancement of infection control practices in surgery, SSI contributes to a substantial burden of morbidity and mortality. The emergence of gut bacilli as the predominant causative agent of SSI is indicative of the need for effective infection-control practices. A paradigm shift of bacteriological profile from Gram-positive bacteria to multidrug-resistant Gram-negative bacilli is a great threat to patient outcome and a major hindrance to progress in the field of surgery. Knowledge of the microbiology of surgical infections and regular surveillance with the feedback of appropriate data to surgeons can be an important component of strategies to reduce SSI risk.
